# Pulmonary embolism complicating *Mycoplasma pneumoniae* pneumonia in children: a retrospective case series

**DOI:** 10.3389/fped.2025.1703027

**Published:** 2026-01-14

**Authors:** Pei Tao, Zhigang Wang, Kaiyu Zhou, Ying Wu

**Affiliations:** 1 Chengdu Women and Children's Central Hospital, School of Medicine, University of Electronic Science and Technology of China, Chengdu, Sichuan, China; 2 Department of Pediatric Pulmonology and Immunology, West China Second University Hospital, Sichuan University, Chengdu, China; 3 Department of Pediatric Caidiology, West China Second University Hospital, Sichuan University, Chengdu, China

**Keywords:** anticoagulation, children, computed tomography pulmonary angiography, *mycoplasma pneumoniae* pneumonia, pulmonary embolism, severe pneumonia

## Abstract

**Objectives:**

To describe the clinical characteristics, management, and outcomes of children with *Mycoplasma pneumoniae* pneumonia (MPP) complicated by pulmonary embolism (PE).

**Methods:**

We conducted a retrospective review of eight children diagnosed with *Mycoplasma pneumoniae* pneumonia complicated by pulmonary embolism between January 2023 and December 2024 at our hospital. The diagnosis of pulmonary embolism was confirmed by computed tomography pulmonary angiography (CTPA). Demographic characteristics, clinical manifestations, laboratory findings, imaging features, treatment strategies, and outcomes were systematically collected.

**Results:**

The cohort included six males and two females, with a mean age of 7.81 ± 3.64 years. The median interval from pneumonia onset to PE diagnosis was 14 days. All patients had severe or refractory MPP. Common symptoms included chest pain (*n* = 6), hemoptysis (*n* = 4), and dyspnea (*n* = 2). CTPA demonstrated pulmonary arterial filling defects in all cases. All patients received anticoagulation therapy with low-molecular-weight heparin followed by rivaroxaban, resulting in favorable clinical outcomes. During 3–6 months of follow-up, complete resolution of emboli was observed, thrombophilia-related laboratory abnormalities normalized, and no recurrence occurred.

**Conclusions:**

Early diagnosis and timely anticoagulation are crucial for favorable outcomes in children with MPP-related PE. Although the small sample size limits generalizability, this case series provides a structured clinical dataset that captures demographic, laboratory, immunological, and imaging features. These data may serve as a reference for future studies aiming to better understand host susceptibility and immunothrombotic mechanisms in pediatric PE.

## 1 Introduction

*Mycoplasma pneumoniae* pneumonia (MPP) is the most common cause of community-acquired pneumonia in children aged 5 years and older in China (1). Severe *M. pneumoniae* pneumonia (SMPP) is defined as MPP meeting the criteria for severe community-acquired pneumonia and is often associated with significant intrapulmonary and extrapulmonary complications (2, 3). Common intrapulmonary complications include plastic bronchitis, moderate-to-large pleural effusion, extensive lung consolidation, pulmonary necrosis, and pulmonary embolism (PE) (4–6). Patients may present with clinical symptoms such as shortness of breath, chest pain, or hemoptysis (7, 8). In addition, extrapulmonary complications can involve multiple organ systems, including the liver, skin, nervous system, and hematologic system (1). PE, although relatively rare in children, can be life-threatening if not diagnosed and treated in a timely manner (9, 10).

Refractory *M. pneumoniae* pneumonia (RMPP) is diagnosed when children continue to experience persistent fever, progressive clinical deterioration, radiological worsening, or extrapulmonary complications despite receiving at least seven days of appropriate macrolide therapy. In recent years, the incidence of both SMPP and RMPP has increased, particularly during epidemic outbreaks, raising concerns regarding severe complications and atypical disease courses (11–15).

Beyond direct pulmonary involvement, *M. pneumoniae* infection can trigger a wide range of extrapulmonary complications through immune-mediated and vascular mechanisms, including autoimmunity, immune complex deposition, endothelial injury, and thrombosis (16–19). These mechanisms may contribute to thromboembolic events such as PE, highlighting the complex interplay between infection, inflammation, and coagulation. However, existing pediatric reports of PE complicating MPP are largely limited to isolated cases or small series, and systematic clinical descriptions remain scarce.

Comprehensive characterization based on structured clinical, laboratory, and imaging data may improve recognition of pulmonary embolism in children with severe or refractory *Mycoplasma pneumoniae* pneumonia. In this study, we retrospectively analyzed a series of pediatric patients with MPP complicated by PE, focusing on clinical presentation, diagnostic evaluation, treatment strategies, and outcomes. This clinically grounded overview may facilitate hypothesis generation and support future integrative research in pediatric thromboembolic disease.

## 2 Methods

### 2.1 Study design and participants

This was a retrospective observational study conducted at the Respiratory Department of Chengdu Women and Children's Central Hospital. Eight pediatric patients diagnosed with MPP complicated by PE were included. The study was conducted from January 2023 to December 2024. Eligible patients were identified by applying strict inclusion and exclusion criteria to the hospital's electronic medical records system.

### 2.2 Ethical statement

The study protocol was approved by the Ethics Committee of Chengdu Women and Children's Central Hospital {Approval No. [2024(95)]}. Written informed consent for computed tomography pulmonary angiography (CTPA) and data use for research purposes was obtained from the legal guardians of all patients. All clinical data were anonymized prior to analysis to ensure patient confidentiality.

### 2.3 Inclusion and exclusion criteria

Inclusion criteria for the PE group were as follows:

Diagnosis of MPP confirmed by: Positive *M. pneumoniae*-specific IgM (titer ≥ 1:160); and/or positive *M. pneumoniae* DNA detection via PCR from nasopharyngeal swabs.
Diagnosis of PE confirmed via CTPA showing embolic occlusion in the pulmonary vasculature.
Informed consent obtained for the CTPA procedure.

The exclusion criteria included:

Alternative causes of PE, such as air embolism, congenital heart disease, central venous catheterization, nephrotic syndrome, surgery, malignancy, or use of hormonal contraceptives in adolescent females.
Incomplete clinical, laboratory, and imaging data.

### 2.4 Microbiological testing

Within 24 h of admission, all patients underwent comprehensive pathogen screening:

*M. pneumoniae* DNA detection from nasopharyngeal swabs using a PCR kit (Tian Long Tech, China; detection limit: 100 copies/ml).
A multiplex respiratory PCR panel targeting influenza A/B, respiratory syncytial virus, adenovirus, human metapneumovirus, and parainfluenza virus (Sansure Biotech Inc.).
Serological IgM antibody testing against *M. pneumoniae*, *Chlamydia pneumoniae, Legionello pneumophila, Rickettsia*, and common respiratory viruses using ELISA kits (Mike Bio, China).
Tuberculosis screening (T-SPOT.TB or PPD), and blood and sputum cultures for bacterial pathogens.

### 2.5 Laboratory and imaging data collection

Clinical and diagnostic data were extracted from electronic medical records and organized into a structured multi-dimensional dataset to enable potential future computational analyses.

#### 2.5.1 Demographic and clinical characteristics

Data included patient age, sex, prior history of venous thromboembolism (VTE) or cardiovascular disease, duration from symptom onset to CTPA diagnosis, intensive care unit (ICU) admission, primary clinical manifestations, and anatomical distribution of PE.

#### 2.5.2 Hematological and coagulation parameters

Complete blood counts and coagulation profiles were reviewed, including white blood cell (WBC) count, absolute neutrophil and lymphocyte counts, hemoglobin (Hb), platelet (PLT) count, plasma D-dimer levels.

#### 2.5.3 Inflammatory and biochemical markers

Inflammatory indices such as C-reactive protein (CRP), procalcitonin (PCT) were recorded. Biochemical data included lactate dehydrogenase (LDH), alanine aminotransferase (ALT), aspartate aminotransferase (AST), total bilirubin, albumin, serum creatinine, blood urea nitrogen (BUN), and serum electrolytes.

#### 2.5.4 Immunological assessment

Serum immunoglobulin levels (IgA, IgG, and IgM), complement components (C3 and C4), and autoimmune markers [antinuclear antibodies (ANAs), extractable nuclear antigens (ENAs)] were analyzed. Lymphocyte subsets (CD3^+^, CD4^+^, CD8^+^, B cells, and NK cells) were evaluated using flow cytometry.

#### 2.5.5 Imaging studies

Radiological investigations included CTPA to determine the presence and distribution of emboli, chest computed tomography or radiography to assess pulmonary involvement, and Doppler ultrasound of the lower extremities when clinically indicated.

### 2.6 Data presentation

Categorical variables were expressed as absolute numbers and proportions (%), while continuous variables were reported as medians and interquartile ranges (IQR) owing to their non-parametric distribution. No formal statistical analyses were performed, and all data are presented descriptively.

## 3 Results

### 3.1 Baseline characteristics

A total of eight children with severe or refractory *M. pneumoniae* pneumonia (SMPP/RMPP) complicated by pulmonary embolism (PE) were included. The cohort comprised six males and two females, aged 2–15 years (median 7.81 ± 3.64 years). Coinfections included *Streptococcus pneumoniae* (*n* = 2), *Escherichia coli* (*n* = 1), and *influenza A* virus (*n* = 1). The median interval from MPP onset to PE diagnosis was 14 days. No patients had a family history of thrombophilia or hereditary disease (Table 1). Fever and cough were observed in all eight patients. Additional symptoms included chest pain (*n* = 6), hemoptysis (*n* = 4), and dyspnea (*n* = 2). Heterogeneous pulmonary involvement and embolic distribution were noted, with a right-lung predominance (Table 1).

**Table 1:** Clinical characteristics of children with Mycoplasma pneumoniae pneumonia complicated by pulmonary embolism.

Case	Age (years)	Gender	Main symptoms	Etiology	Lung consolidation site	Embolic site	Complications
1	2.75	M	Cough, fever, dyspnea	*M. pneumoniae*	Right upper and lower lobes	Right upper, middle, and lower lobes	Kawasaki disease; Necrotizing pneumonia
2	4.75	M	Cough, fever, dyspnea	*M. pneumoniae, Streptococcus pneumoniae*	Right upper lobe; left lower lobe	Both lower lobes	Necrotizing pneumonia
3	8	M	Cough, fever, chest pain, hemoptysis	*M. pneumoniae, S. pneumoniae*	Right lower lobe; left upper and lower lobes	Both lower lobes; left upper lobe	Necrotizing pneumonia
4	9	M	Cough, fever, chest pain, hemoptysis	*M. pneumoniae*	Right lower lobe	Right lower lobe	Necrotizing pneumonia
5	9	M	Cough, fever, chest pain, hemoptysis	*M. pneumoniae*	Right lower lobe	Right lower lobe	Necrotizing pneumonia
6	6	F	Cough, fever, chest pain	*M. pneumoniae*	Bilateral lower lobes	Right lower pulmonary artery; anterior and posterior basal segments (right)	Necrotizing pneumonia
7	8	F	Cough, fever, chest pain	*M. pneumoniae, Escherichia. coli*	Right lower lobe	Right lower lobe	None
8	15	M	Cough, fever, chest pain, hemoptysis	*M. pneumoniae, Influenza A*	Left lower lobe	Left lower lobe	Necrotizing pneumonia

M, male; F, female.

### 3.2 Laboratory, immunological, and coagulation profiles

Elevated D-dimer levels were observed in 7 of the 8 patients at the time of PE diagnosis, with a median of 4.695 mg/L (IQR 1.725–9.105 mg/L). One patient had a level within the normal range (0.19 mg/L). Median WBC was 8.98 × 10⁹/L (IQR 8.09–12.79), and median platelet count was 277 × 10⁹/L (IQR 238–323). Median CRP and procalcitonin levels were 21.5 mg/L (IQR 13.35–34.9) and 0.121 ng/ml (IQR 0.103–0.291), respectively. LDH was elevated in five patients (median 361.8 U/L, IQR 233.9–482.4). Immunologic evaluation showed IgG 8.095 g/L (IQR 7.425–9.280), IgA 1.515 g/L (IQR 1.175–1.910), IgM 1.285 g/L (IQR 1.230–1.425), complement C3 1.375 g/L (IQR 1.246–1.500), and C4 0.320 g/L (IQR 0.266–0.334). Lymphocyte subsets were within reference ranges: CD3 + 62.84% (IQR 56.33–69.48), CD4 + 29.27% (IQR 25.47–38.17), CD8 + 26.45% (IQR 22.03–32.20), B cells 16.67% (IQR 15.23–19.71), and NK cells 15.38% (IQR 9.79–19.28). No abnormalities were detected in thrombophilia-related tests, including antiphospholipid antibodies, protein C and S, coagulation factors, or inhibitors (Table 2).

**Table 2:** Laboratory findings of children with Mycoplasma pneumoniae pneumonia complicated by pulmonary embolism.

Parameter	Case 1	Case 2	Case 3	Case 4	Case 5	Case 6	Case 7	Case 8	Median (IQR)
WBC (×10⁹/L)	7.7	30.07	5.92	9.16	8.48	16.22	9.36	8.8	8.98 (8.09–12.79)
PLT (×10⁹/L)	300	711	201	323	254	241	323	235	277 (238–323)
CRP (mg/L)	27	18.5	157.6	24.5	7.8	17.8	42.8	8.9	21.5 (13.35–34.9)
PCT (ng/ml)	0.127	0.107	0.412	0.047	0.100	0.115	0.550	0.170	0.121 (0.103–0.291)
D-dimer (mg/L)	10.05	8.16	5.32	4.07	0.81	2.64	19.61	0.19	4.695 (1.725–9.105)
LDH (U/L)	910.1	425.2	214	539.6	221	368.2	355.4	246.8	361.8 (233.9–482.4)
IgG (g/L)	7.56	6.87	7.58	9.36	9.04	8.61	11.04	7.38	8.095 (7.425–9.280)
IgA (g/L)	1.82	1.01	1.25	1.94	1.38	1.65	2.18	1.15	1.515 (1.175–1.910)
IgM (g/L)	1.33	1.23	1.24	1.04	1.78	1.41	1.43	1.23	1.285 (1.230–1.425)
C3 (g/L)	1.52	1.325	1.146	1.462	1.249	1.513	1.425	1.245	1.375 (1.246–1.500)
C4 (g/L)	0.324	0.206	0.317	0.253	0.342	0.337	0.308	0.327	0.320 (0.266–0.334)
CD3^+^ (%)	55.32	49.47	67.23	78.13	62.24	63.44	59.36	70.23	62.84 (56.33–69.48)
CD4^+^ (%)	31.78	25.71	36.34	42.24	26.76	21.94	25.39	38.78	29.27 (25.47–38.17)
CD8^+^ (%)	32.69	19.44	22.32	30.71	21.93	33.27	24.68	28.22	26.45 (22.03–32.20)
B cells (%)	15.21	36.85	15.28	16.65	8.76	16.68	20.10	18.53	16.67 (15.23–19.71)
NK cells (%)	8.66	13.16	15.35	4.57	26.28	19.10	15.40	19.34	15.38 (9.79–19.28)

WBC, white blood cell count; PLT, platelet count; CRP, C-reactive protein; PCT, procalcitonin; LDH, lactate dehydrogenase; IgG/IgA/IgM, immunoglobulins; C3/C4, complement components; NK, natural killer cells. Data are presented as median (IQR). Reference ranges: WBC, 3.6–13.0 × 10⁹/L; PLT, 125– 462 × 10⁹/L; CRP, <10 mg/L; PCT, <0.05 ng/mL; D-dimer, <0.50 mg/L FEU; LDH, 120–250 U/L; IgG, 6.7–15.3 g/L; IgA, 0.52–2.74 g/L; IgM, 0.48–2.31 g/L; C3, 0.7–1.4 g/L; C4, 0.1–0.4 g/L; CD3^+^, 60%–80%; CD4^+^, 26.17–40.76%; CD8^+^,19.08–34.06%; B cells (CD19^+^), 9–22.24%; NK cells (CD16^+^/CD56^+^), 10.21–20.12%.

The institutional reference ranges for all laboratory parameters are provided in the footnote of Table 2.

### 3.3 Imaging examination

**Figure 1:**
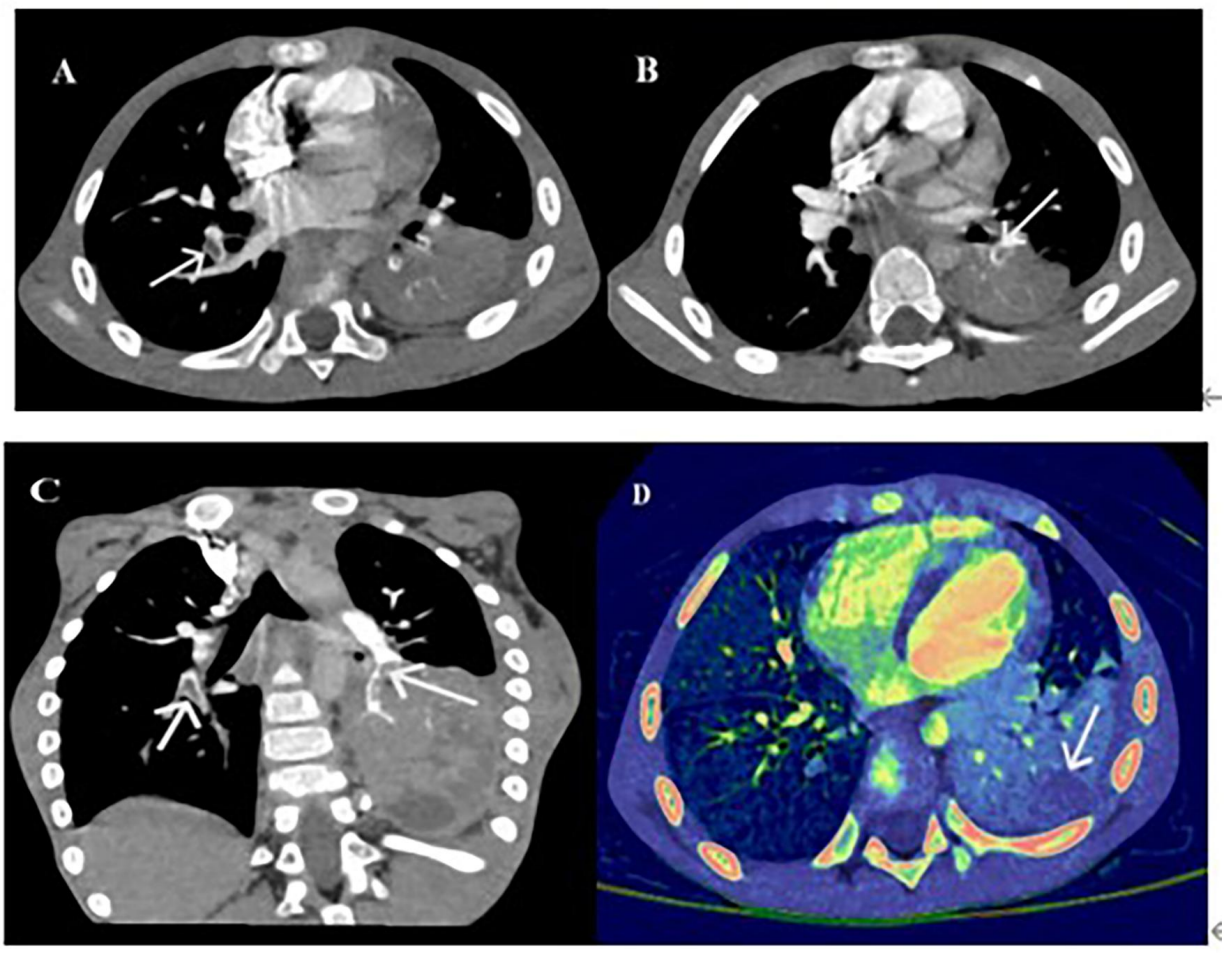
Computed tomography pulmonary angiography (CTPA): **(A)** Right pulmonary artery embolism. **(B)** Left pulmonary embolism (PE). **(C)** Double PE coronal axis diagram. **(D)** Energy spectrum of double PE.

**Figure 2:**
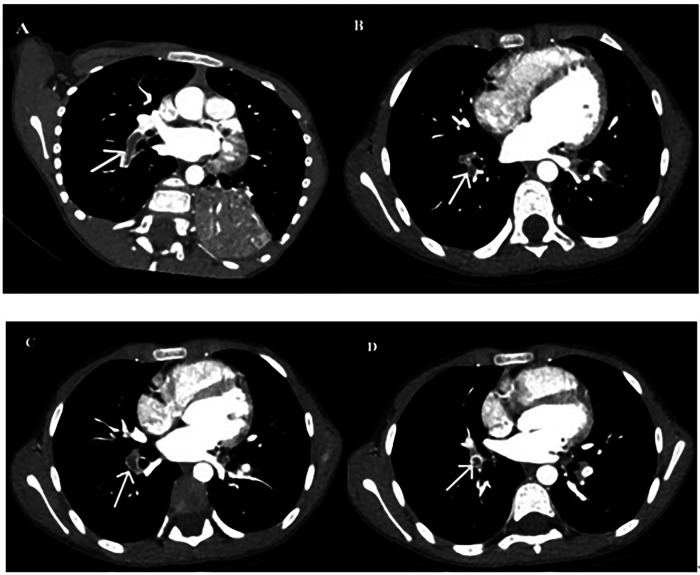
**(A–D)** Left PE observed at different time points.

**Figure 3:**
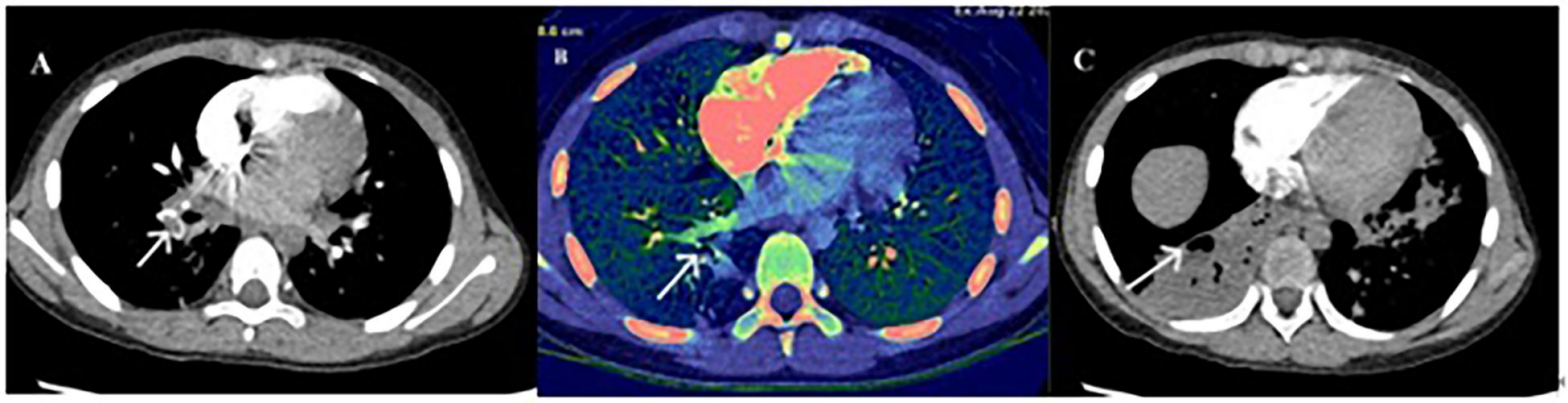
CTPA: **(A)** pulmonary thrombosis. **(B)** Angiography and energy spectrum. **(C)** Corresponding necrotic areas after PE.

Computed tomography pulmonary angiography (CTPA) confirmed PE in all eight cases. Emboli were located in the right lung (*n* = 5), left lung (*n* = 1), or both lungs (*n* = 2). PE-related vascular occlusion was associated with localized parenchymal necrosis in affected regions. Five patients developed necrotizing pneumonia, and one had Kawasaki disease (Figures 1–3).

### 3.4 Treatment and follow-up

**Figure 4:**
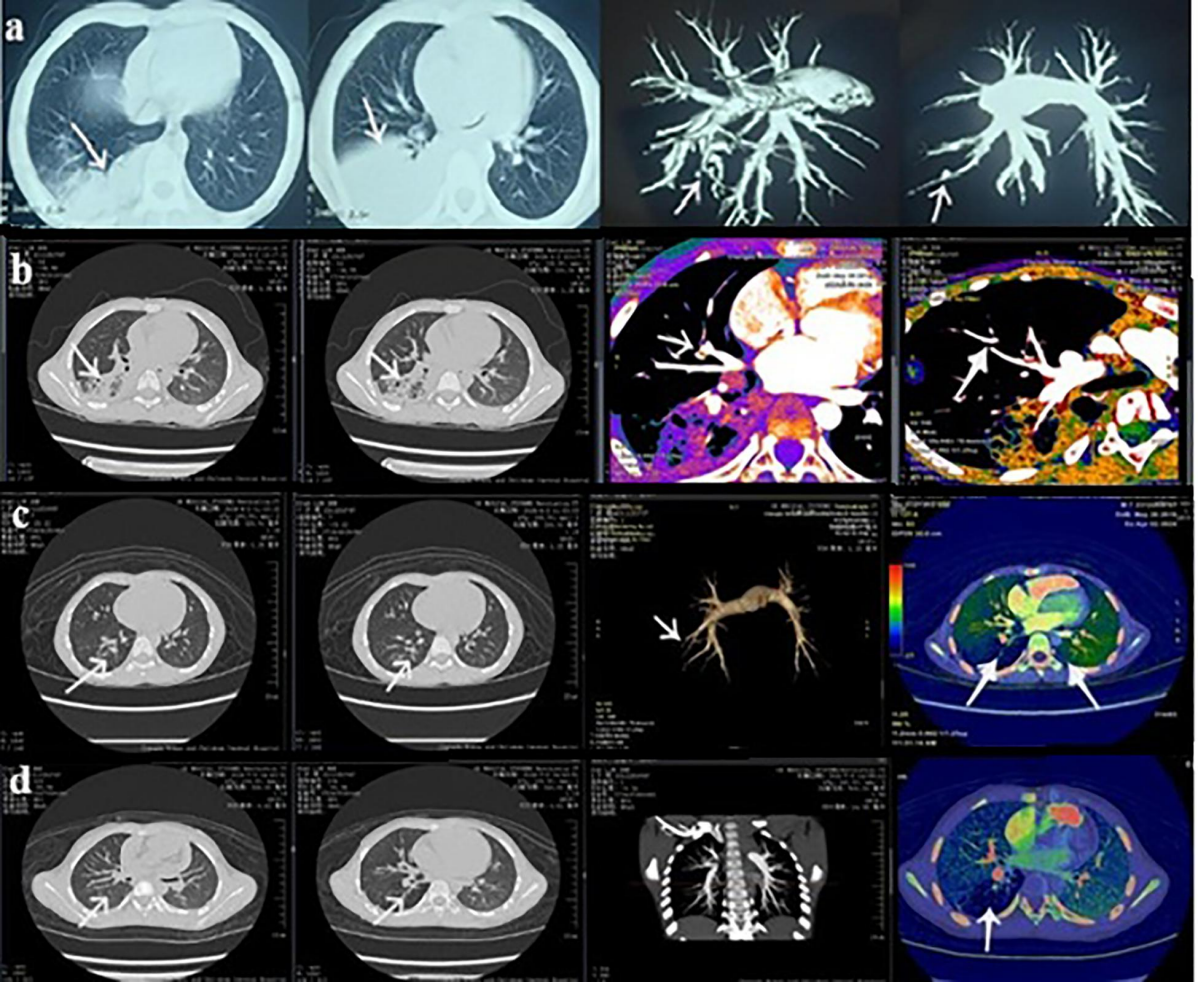
CPTA (Case no. 7): **(a)** Baseline. **(b)** 1-month follow-up. **(c)** 3-month follow-up. **(d)** 6-month follow-up.

All patients received enoxaparin during hospitalization (7–10 days). Outpatient oral anticoagulation with rivaroxaban was administered in six cases (3–6 months). Anti-*M. pneumoniae* therapy included erythromycin, azithromycin, doxycycline, and/or cefotaxime. One patient received antiviral therapy (peramivir). All eight patients demonstrated complete thrombus resolution during follow-up (3–6 months), with no recurrence observed (Table 3, Figure 4).

**Table 3:** Treatment and follow-up.

Case	Antibiotics	Anticoagulant		Outcomes	
		Hospitalization	Outpatient	3M	6M
1	Erythromycin, Azithromycin	Enoxaparin for 7 d	Rivaroxaban for 3 m	Disappeared	–
2	Azithromycin, Cefotaxime	Enoxaparin for 7 d	Rivaroxaban for 3 m	Disappeared	–
3	Doxycycline, Cefotaxime	Enoxaparin for 7 d	Rivaroxaban for 3 m	Disappeared	–
4	Doxycycline	Enoxaparin for 7 d	Rivaroxaban for 3 m	Disappeared	–
5	Erythromycin	Enoxaparin for 7 d	–	Disappeared	–
6	Erythromycin	Enoxaparin for 7 d	Rivaroxaban for 6 m	–	Disappeared
7	Cefotaxime, Azithromycin	Enoxaparin for 10 d	Rivaroxaban for 6 m	–	Disappeared
8	Doxycycline, Peramivir	Enoxaparin for 7 d	–	Disappeared	–

M, months; d, days.

## 4 Discussion

Pulmonary embolism (PE) is a relatively rare but potentially life-threatening complication in children, especially when it occurs secondary to severe or refractory *Mycoplasma pneumonia* (MPP) (9, 20–26). The epidemiological data for the pediatric population in China is still limited, although recent reports indicate an upward trend. In our study cases, PE occurred within the median 14 days after the onset of MPP, highlighting the importance of early identification of severely or refractory diseases in children.

Previous studies have reported the positive rate of thrombotic autoantibodies in children with MPP-related embolic events (27). In contrast, our cohort did not demonstrate significant abnormalities in thrombosis-related autoantibodies, including antiphospholipid antibodies and antinuclear antibodies, suggesting heterogeneity in immune-mediated thrombotic mechanisms across pediatric populations.

*M. pneumoniae* infection activates B cell–mediated immune responses, leading to increased IgM and IgG production and alterations in IgA and IgE levels. Severe MPP has also been associated with pathological lymphocyte activation, elevated complement components C3 and C4, and dysregulated T cell subsets, including reduced CD3^+^/CD4^+^ and NK cells with increased CD8^+^ T cells (28–31). Although elevated IgA has been implicated in the progression of pediatric PE (32), such immune abnormalities were not observed in our cohort. These discrepancies may reflect population-specific immune characteristics, underlying genetic heterogeneity, or the limited sample size of the present study. Nevertheless, the systematic documentation of immune parameters alongside clinical and imaging findings in our series provides a clinically grounded framework for future studies exploring immune–thrombotic mechanisms in MPP-related PE.

The clinical manifestations of pediatric pulmonary embolism are diverse and often atypical. In our case series, 6 patients experienced chest pain, 4 had hemoptysis, and 2 had breathing difficulties. Laboratory results also showed heterogeneity: 7 patients had elevated D-dimer levels (≥0.5 mg/L), while platelet count, CRP, and LDH were elevated to varying degrees. However, D-dimer alone is insufficient to rule out PE in children, as normal values may be observed in confirmed cases. These indicators were associated with disease severity and necrotizing pneumonia (33–41). Taken together, these observations indicate the interaction between inflammation, coagulation, and pediatric MPP-related PE lung injury, and highlight the need for imaging evaluation, such as CTPA, in severe or refractory cases.

Computed tomography pulmonary angiography (CTPA) remains the diagnostic gold standard for pulmonary embolism (PE) (42). In pediatric patients, its use is often limited by concerns regarding radiation exposure, small vessel caliber, and nonspecific clinical presentations (43). Despite these challenges, CTPA played a central role in our cohort by confirming the diagnosis of PE in all eight patients and enabling dynamic assessment of thrombus resolution during follow-up. Notably, pulmonary necrotic changes were observed in the disease progression of several children, raising questions about persistent perfusion defects and potential microvascular damage. These imaging results provide in-depth insights into the evolution of pediatric PE under anticoagulation therapy and emphasize the importance of careful radiological assessment. Despite the limitations of sample size and follow-up time, our longitudinal imaging observations highlight the unresolved issues regarding vascular changes after thrombosis, and this requires further study in a larger pediatric cohort.

The management of thromboembolic diseases in children still mainly relies on consensus recommendations and inferences from adult studies (44–46). In our study cases, all patients initially received low-molecular-weight heparin anticoagulation therapy, and most patients subsequently switched to rivaroxaban for maintenance treatment. The clinical outcomes were good, and complete resolution of pulmonary embolism was observed during the follow-up period. It is notable that both patients who continued oral anticoagulation treatment and those who stopped treatment early showed improvement, highlighting the current uncertainty.

In summary, the case series we studied provided a rare and clinically detailed group sample for children with pulmonary embolism related to MPP. By highlighting new observations, including immune heterogeneity, atypical symptom manifestations, and differences in laboratory results, these findings complement the existing literature and point out the areas that require further research. Despite limitations such as the retrospective design, small sample size, and single-center setting, this dataset lays the foundation for prospective, multicenter studies to elucidate the natural course, risk factors, and long-term prognosis of pediatric pulmonary embolism, and may provide guidance for targeted clinical management strategies.

## 5 Conclusion

Pulmonary embolism (PE) complicating *M. pneumoniae* pneumonia (MPP) is an uncommon but clinically serious condition in children (47, 48). In this retrospective case series, we described eight pediatric patients with MPP-related PE, systematically summarizing their clinical presentation, laboratory and immunological findings, imaging characteristics, management strategies, and outcomes. Although the small sample size limits the generalizability of our observations, the rarity of this complication makes detailed case-based data clinically informative. Our findings highlight the diagnostic challenges of PE in pediatric MPP and underscore the importance of timely recognition and appropriate anticoagulant therapy. This structured clinical experience may serve as a reference for clinicians and provide a foundation for future studies aimed at improving risk assessment and management of pediatric thromboembolic disease.

## Data Availability

The original contributions presented in the study are included in the article/Supplementary Material, further inquiries can be directed to the corresponding author.
